# Phosphodiesterase 5 Attenuates the Vasodilatory Response in Renovascular Hypertension 

**DOI:** 10.1371/journal.pone.0080674

**Published:** 2013-11-15

**Authors:** Johannes Stegbauer, Sebastian Friedrich, Sebastian A. Potthoff, Kathrin Broekmans, Miriam M. Cortese-Krott, Ivo Quack, Lars Christian Rump, Doris Koesling, Evanthia Mergia

**Affiliations:** 1 Klinik für Nephrologie, Universitätsklinikum Heinrich-Heine-Universität Düsseldorf, Düsseldorf, Germany; 2 Klinik für Kardiologie, Universitätsklinikum Heinrich-Heine-Universität Düsseldorf, Düsseldorf, Germany; 3 Institut für Pharmakologie Ruhr-Universität Bochum, Bochum, Germany; University of Bonn, Germany

## Abstract

NO/cGMP signaling plays an important role in vascular relaxation and regulation of blood pressure. The key enzyme in the cascade, the NO-stimulated cGMP-forming guanylyl cyclase exists in two enzymatically indistinguishable isoforms (NO-GC1, NO-GC2) with NO-GC1 being the major NO-GC in the vasculature. Here, we studied the NO/cGMP pathway in renal resistance arteries of NO-GC1 KO mice and its role in renovascular hypertension induced by the 2-kidney-1-clip-operation (2K1C). In the NO-GC1 KOs, relaxation of renal vasculature as determined in isolated perfused kidneys was reduced in accordance with the marked reduction of cGMP-forming activity (80%). Noteworthy, increased eNOS-catalyzed NO formation was detected in kidneys of NO-GC1 KOs. Upon the 2K1C operation, NO-GC1 KO mice developed hypertension but the increase in blood pressures was not any higher than in WT. Conversely, operated WT mice showed a reduction of cGMP-dependent relaxation of renal vessels, which was not found in the NO-GC1 KOs. The reduced relaxation in operated WT mice was restored by sildenafil indicating that enhanced PDE5-catalyzed cGMP degradation most likely accounts for the attenuated vascular responsiveness. PDE5 activation depends on allosteric binding of cGMP. Because cGMP levels are lower, the 2K1C-induced vascular changes do not occur in the NO-GC1 KOs. In support of a higher PDE5 activity, sildenafil reduced blood pressure more efficiently in operated WT than NO-GC1 KO mice. All together our data suggest that within renovascular hypertension, cGMP-based PDE5 activation terminates NO/cGMP signaling thereby providing a new molecular basis for further pharmacological interventions.

## Introduction

Hypertension is the leading risk factor for cardiovascular mortality and has been associated with alterations in vascular relaxation. One of the major pathways that mediate vascular relaxation is the NO/cGMP signaling cascade, in which the NO-stimulated guanylyl cyclase (NO-GC) holds a key position by translating the NO signal into cGMP formation [1–3]. The NO-GC contains a prosthetic heme group as a specific NO binding site and exhibits enhanced rates of cGMP formation in response to NO activation [4]. Two isoforms of the NO receptor GC exist; NO-GC1 and NO-GC2 which consist of an identical β subunit dimerized to an α_1_ or α_2_ subunit, respectively [5]. Knock-out (KO) mice deficient in either one of the NO-GCs, NO-GC1 or NO-GC2, revealed that both NO-GCs participate in vascular relaxation [6]. Outside the central nervous system, NO-GC1 is the major isoform, particularly in aorta, where NO-GC1 represents approximately 90% of the WT NO-GC content. Although deletion of the NO-GC1 resulted in reduced vascular relaxation, the NO-GC1 KO mice develop hypertension only on the 129S6 background which displays higher activity of the renin-angiotensin-aldosterone system (RAAS) than other inbred mouse strains [7]. 

In the vasculature, the cGMP-mediated relaxation is transduced by the cGMP-dependent protein kinase I (PKGI) [8]. The cGMP effects are controlled by the activity of cGMP-hydrolysing phosphodiesterases (PDEs) with PDE1 and PDE5 being of particular importance for cGMP degradation in vascular smooth muscle [9]. PDE1 is stimulated by Ca^2+^/calmodulin and has been suggested to play a more dominant role under conditions of higher calcium (Ca^2+^), e.g., in the presence of vasoconstrictors. Under conditions of low calcium, PDE5 is considered to be the major cGMP-degrading PDE. NO-induced cGMP increase does not only activate PKGI but also cause allosteric activation of PDE5 by cGMP binding to the regulatory GAF-A domain of PDE5 which is paralleled by PKGI-mediated phosphorylation [10,11]. Hence, by activating PDE5 the NO/cGMP signal initiates a negative feedback loop to limit its own action [12].

It is widely accepted that the vasodilatory NO/cGMP pathway counteracts the vasoconstriction induced by the renin-angiotensin system (RAS) to maintain normal circulatory homeostasis. Thus, an activated RAS as in the 2-kidney 1-clip (2K1C) Goldblatt model of human renovascular hypertension causes hypertension and a reduction of endothelium-dependent relaxation [13–16].

In the present study, we characterized the NO/cGMP pathway in kidneys of NO-GC1 KO mice, in which the major NO-GC isoform of the kidney, NO-GC1, is deleted. Despite a pronounced reduction of cGMP forming activity (80%), the decrease of the renal cGMP content was moderate (50%) as was the decrease of relaxation of the renal vasculature determined in the isolated perfused kidneys. Comparison of endothel- and smooth muscle-dependent relaxation indicated increased NO formation in the NO-GC1 KO that partially compensates for the deficiency of NO-GC1. 

We challenged NO-GC1 KO mice by the 2K1C operation to investigate the impact of the NO/cGMP pathway on renovascular hypertension. Unexpectedly, 2K1C-operated NO-GC1 KO mice did not develop a higher degree of hypertension than WT and the reduction of endothelium-dependent relaxation found in operated WT kidney and aortas was not observed in operated NO-GC1 KO mice. Furthermore, a reduction of responsiveness towards exogenous NO was detected in 2K1C WT that was not based on decreased NO-GC content or altered sensitivity towards cGMP. As the reduced NO sensitivity in operated WT kidneys was restored by the inhibitor of PDE5, sildenafil, PDE5 emerges as the key vascular component responsible for the reduction of endothelium-dependent relaxation in renovascular hypertension. 

## Materials and Methods

### KO mice

Studies were performed with NO-GC1 KO mice lacking the α_1_ subunit of the heterodimeric NO-GC1 receptor and wild-type (WT) littermates backcrossed to C57Bl/6Rj background for 5-7 times (N5-N7 generation). Generation and genotyping of the NO-GC1 KO mice have been described by Mergia et al., 2006 [6]. Two- to 4-month-old males (~30 g) were used in all studies. Mice were fed a low salt diet containing 0.12% NaCl (Sniff, Soest, Germany) and allowed free access to tap water. All animal investigations conformed to the Guide for the care and Use of laboratory Animals published by the US national Institutes of Health (NIH Publication No. 85-23, revised 1996) and were also approved by the local animal care committee (Landesamt für Natur, Umwelt und Verbraucherschutz Nordrhein-Westfalen, Recklinghausen, licence no. AZ. 8.87-50.10.34.08.216). 

### 2K1C operation

In the 2K1C model, partial occlusion of one renal artery reduces renal blood flow causing activation of the RAS. The 2K1C operation was performed in 8 week old male mice according to the reported method [17] with some modifications. In brief, mice were anesthetized with Ketamine (100 mg/kg, i.p.) and Xylazin (5 mg/kg, i.p.). The depth of anaesthesia was confirmed by lack of toe pinch response. After a latero-abdominal incision, the left renal artery dissected from renal vein and nerves over a short segment close to the abdominal aorta was partially occluded (silver clip, 0.12mm internal gap, Klaus Effenberger, Med. Tech. Gerätebau, Pfaffing, Germany). Mice with an 80 to 90% reduction of renal blood flow after clipping measured with a laser Doppler blood flow probe (ADI Instruments) were used. Sham-operated mice, which underwent the same surgical procedure except for placement of the renal artery clip, served as controls. 

### Isolated perfused murine kidneys

Experiments with isolated perfused kidneys were performed using the non-clipped kidney 4 weeks after 2K1C or sham operation according to the method described previously [18]. Perfusion pressure was monitored continuously with a Statham P23 Db pressure transducer (Gould, Oxnard, CA) coupled to a Watanabe pen recorder (Graphtec Corp., Tokyo, Japan). Changes in perfusion pressure reflected changes in vascular resistance with an increase indicating vasoconstriction. Renal vasoconstriction was induced by norepinephrine (NA 1 µM; Sigma-Aldrich). Vasorelaxation induced by carbachol (30 µM; Sigma-Aldrich) was recorded in presence of diclofenac (3 µM). Concentration-response curves of the vasodilators GSNO (Alexis Corp.) and 8-pCPT-cGMP (8-(p-chlorophenylthio)-cGMP; Biolog Inc.) were assessed in the presence of L-NAME (300 µM; Sigma-Aldrich) and diclofenac (3 µM). To test the influence of vinpocetine (10 µM; Calbiochem) and sildenafil (300 nM; a generous gift from Pfizer) on vasodilative properties of GSNO, the respective PDE inhibitors were added to the perfusion solution 10 minutes before GSNO administration. Renal relaxation is expressed as pressure reduction with the pressure of the precontracted kidney set as 100%. 

### Preparation of murine homogenates

Mice were anesthetized by CO_2_ inhalation and decapitated, non-clipped kidneys or aortas (free of surrounding tissue) were removed and homogenized immediately with a glass/glass homogenizer (700 rpm) in buffer (50 mmol/L triethanolamine (TEA)/HCl, 50 mmol/L NaCl, 1 mmol/L EDTA, 2 mmol/L DTT, 0.2 mmol/L benzamidine, 0.5 mmol/L phenylmethylsulfonylfluoride (PMSF), and 1 µmol/L pepstatin A; pH 7.4, 4°C). After centrifugation (800 x g, 5 min, 4°C), homogenates were used in experiments as described. The protein concentration was determined in triplicates and repeated three times (Bradford, Bio-Rad).

### Determination of NO-stimulated GC activity

NO-stimulated GC activity was measured for 10 min (37°C ) in kidney and aorta homogenates (2 µg and 3 µg protein, respectively), in the presence of 100 µM DEA-NO (2-(N,N-diethylamino)-diazenolate-2-oxide, Alexis) as described previously [6].

### Measurement of cGMP-hydrolyzing PDE activity

PDE activity in homogenates was measured by the conversion of [^32^P]cGMP (synthesized from [α-^32^P]GTP using purified NO-GC) to guanosine and [^32^P]phosphate in the presence of alkaline phosphatase (Sigma) at 37 °C for 7 min. Reactions mixtures (0.1 ml) contained 0.5-15 µl of the homogenates (~1-15 µg protein), [^32^P]cGMP (~2 kBq), 1 µM or 30 nM cGMP, 12 mM MgCl_2_, 3 mM DTT, 0.5 mg/ml BSA, 2 U of alkaline phosphatase, and 50 mM TEA/HCl, pH 7.4. Reactions were stopped by adding 900 µl ice cold charcoal suspension (30% activated charcoal in 50 mM KH_2_PO_4_, pH 2.3). After pelleting the charcoal by centrifugation, [^32^P] phosphate was measured in supernatant. Sildenafil 100 nM was used to inhibit PDE5. PDE assays were carried out in triplicates and repeated two or three times.

### Western blot analysis

Separation of proteins (homogenates 50 µg/lane), blotting, detection and quantification of the chemiluminescence signals were performed as described [19]. The signals of the α_2_ and β_1_ subunit of the NO-GCs, eNOS and PDE5 were standardized relative to the amount of the respective protein in the WT sample on the same blot. eNOS phosphorylation was assessed as the ratio of phospho-eNOS and eNOS signals standardized to the one of the WT sample on the same blot. eNOS and phospho-eNOS (Ser1177) antibodies used in a 1:1000 dilution were from BD Biosciences. Antibodies against the α_1_, α_2_ and β_1_ subunits were raised and purified as described [6]. PDE5 antibody used in a 1:1000 dilution was from Cell Signaling Danvers, USA. Beta tubulin antibody used in 1:500 dilution was from Abcam, Cambridge, UK.

### Determination of cGMP content in renal cortical slices

Cortical slices (250 µm) of kidneys were cut with a vibratome (NVSLM1 from WPI), equilibrated for 30 min in temperate (37 °C), oxygenated (in 95% O_2_, 5% CO_2_) Krebs-Henseleit buffer and thereafter were incubated with ODQ (20 µM, 15 min), or Cch (30 µM, 3 min), or DEA-NO (100 µM, 3 min), or sildenafil (100 µM, 10 min). The cGMP levels of equilibrated untreated slices were taken as controls. After incubation, slices were snap frozen in liquid nitrogen, homogenized in 70% (v/v) ice-cold ethanol using a glass/glass homogenizer, and then centrifuged (14,000 x g, 15 min, 4°C). Supernatants were dried at 95°C and the cGMP content was measured in duplicate by RIA. To standardize the different samples, protein pellets were dissolved in 0.1 M NaOH/0.1% SDS, and protein content was determined using the bicinchoninic acid method (Uptima). 

### Determination of NOS activity

Renal homogenates were prepared with a glass/glass homogenizer (1500 rpm, 4°C) in 1 ml buffer (25 mmol/L TRIS-HCl, 10 mmol/L EDTA at pH 7.4, 4°C) supplemented with a commercial cocktail of protease/phosphatase inhibitors (Pierce, Thermo Scientific, Bonn, Germany ) and centrifugated at 1000 x g, 15 min, 4°C. NOS activity of 1 µg total protein was assayed by detecting NO produced by the conversion of L-arginine to citrulline using a NO-specific fluorescent probe 4-Amino-5-Methylamino-2',7'-Difluorofluorescein (DAF-FM) diacetate. Briefly, the reaction was conducted in the dark in a buffer containing 1 µM DAF-FM diacetate, 3 mM L-arginine, 2 μM FAD, 2 μM FMN, 1 mM NADPH, 6 μM tetrahydrobiopterine, 0.04 µg/µl calmodulin, and 20 mM CaCl_2_ for 10 min at RT and green fluorescence (ex 485 nm, em 520 nm) due to the nitrosation of DAF-FM [20] was measured in a FluoStar Omega (BMG Labtech, Ortenberg, Germany). NOS-independent background signal was assessed in all samples by adding the specific NOS inhibitor L-NAME (1 mM). Fluorescence-background was taken as index for NOS-dependent NO production.

### Organ bath experiments with aortic rings

Thoracic aortic rings were mounted isometrically on fixed segment support pins in two four-chamber myographs (Multi Myograph Model 610 M, Danish Myo Technology, Denmark) containing 5 ml of Krebs-Henseleit buffer (118 mmol/L NaCl, 4.7 mmol/L KCl, 1.2 mmol/L MgSO4, 1.2 mmol/L KH_2_PO_4_, 25 mmol/L NaHCO_3_, 2.55 mmol/L CaCl_2_, 7.5 mmol/L D-glucose) in the presence of diclofenac (3 µmol/L) and gassed with 5% CO_2_ in O_2_. Resting tension was set to 5 mN. After equilibration, rings were contracted (1 µmol/L NA) and vasodilation to carbachol (30 µmol/L) was recorded. Each experiment was performed in parallel with four aortic rings each derived from sham- and 2K1C-operated WT or NO-GC1 KO mice, respectively.

### Isolation of preglomerular vessels

Preglomerular vessels containing mainly interlobular arteries and afferent arterioles were isolated by a modified iron oxide-sieving technique as described [21]. As minor modifications, the kidneys were perfused via cannulation of the aorta, and smaller needles (20G, 23G) and pore sieves (100 and 80 µm) were used for separation of tissue and renal particles, respectively. 

### Analysis of PDE5 mRNA content

Preglomerular vessels of the non-clipped kidneys were used to study the PDE5 mRNA content. After homogenization of isolated vessels with a Tissue Ruptor (Qiagen, Germany), total RNA was isolated using a RNA Micro Kit (Qiagen, Germany) according to the manufacturer’s instructions. Quantitative PCR was performed with an ABI PRISM 7300 (Applied Biosystem, Germany) and the SYBR Green master mix (Qiagen, Germany). The PCR reaction was performed in a total volume of 20 µl with 1 µl cDNA corresponding to 100 ng RNA as template and 1 pmol of each PDE5 primer (NM_153422; Qiagen, Germany). The two-step PCR conditions were 2 min at 50°C, 15 min at 95°C, followed by 40 cycles (denaturation of 94°C for 15 s; annealing 55 °C 30 s and extension at 72°C for 34 s). Experiments were performed in triplicate. Glyceraldehyde-3-phosphate dehydrogenase (GAPDH) was chosen as the endogenous control (housekeeping gene). The levels of PDE5 cDNA were normalized to GAPDH by the ∆C_T_ method. 

### Blood pressure measurements

Systolic blood pressures were measured in conscious mice by tail-cuff plethysmography (BP-98A; Softron Co.). For habituation, mice were trained daily for five days. After the training period, 10 measurements per mouse were recorded daily for 5 days, 1 week before and 4 weeks after 2K1C-operation. 

### Oral administration of sildenafil

Sildenafil citrate (100-mg tablets; Viagra; Pfizer) was dissolved in acidified water (pH ~ 3) to a final concentration of 800 mg/L, and given ad libidum, resulting in the ingestion of approximately 100 mg/kg d of sildenafil [22]. The free plasma concentration of sildenafil determined by measuring inhibition of recombinant PDE5 at 1 µM cGMP amounted to 21 ± 5 nM which is around the IC_50_ for PDE5 inhibition (10 nM sildenafil). Mice were treated with sildenafil in the fourth week after the 2K1C-operation.

### Statistical analysis

Data are expressed as mean ± SEM (n=number of animals). Statistical analysis was carried out by the unpaired or paired 2-tailed Student's t test. Concentration-response curves were compared by ANOVA for repeated measurements. Differences were considered significant at a P value of less than 0.05. 

## Results

### Cyclic GMP in kidneys of NO-GC1 KO mice

**Figure 1:**
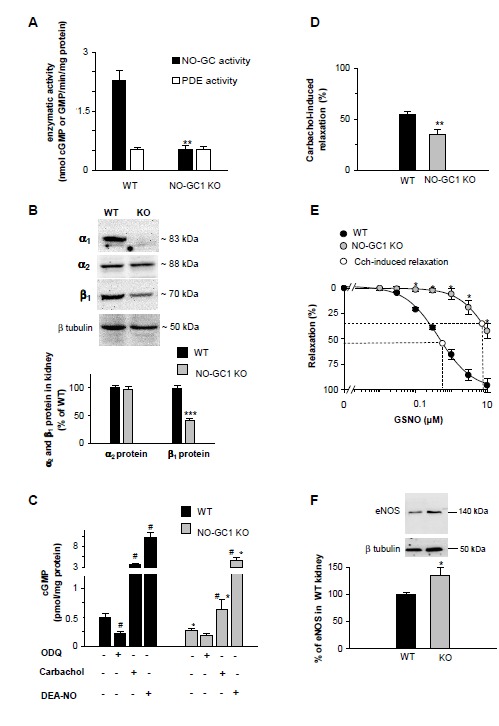
Reduced cGMP response in kidneys of NO-GC1 KO mice. **A**, NO-stimulated cGMP-forming activity (100 µM DEA-NO) determined in kidney homogenates of WT and NO-GC1 KO mice (n=19 and 6 mice, respectively). PDE activity in WT and KO kidneys measured with 1 µM cGMP as substrate (n=11 and 7 mice, respectively). ** P< 0.01 versus WT, unpaired Student's t test. B, absent of the α1 subunit in NO-GC1 KO kidney homogenates and quantification of the α2 and β1 subunit content with respect to the subunit amount in WT using subunit specific antibodies (n=8 mice per genotype). Representative strips with α1, α2, β1 and the respective β tubulin bands of the same lanes are shown above. *** P< 0.001 versus WT, unpaired Student's t test. C, cyclic GMP content determined in renal cortical slices of WT and KO mice without any addition (n=20 and 10 mice, respectively), or in the presence of ODQ (20 µM, 15 min; n=5 and 4 mice, respectively) or carbachol (30 µM, 3 min; n=10 and 6 mice, respectively) or DEA-NO (100 µM 3 min; n=10 and 7 mice, respectively). # P< 0.01 versus untreated slices in the same group; *P< 0.01 versus WT, unpaired Student's t test. D, carbachol-induced relaxation of NA-contracted isolated perfused kidneys of NO-GC1 KO (n=12) and WT mice (n=21). ** P< 0.01 versus WT, unpaired Student's t test. E, concentration-response curves for GSNO-induced relaxation of NA-contracted isolated perfused kidneys in the presence of L-NAME (n=5 WT and 7 KO mice). The white dots indicate the Cch-induced relaxation plotted on the curves to determine the corresponding GSNO concentration. F, Western blot detection of eNOS in 50 µg kidney homogenates of NO-GC1 KO (n=8) and WT mice (n=5) and quantification with respect to the eNOS amount in WT kidneys. A representative strip with eNOS and the respective β tubulin bands of the same lanes is shown above. * P< 0.05 versus WT, unpaired Student's t test.

Deletion of the NO-GC1 isoform resulted in 80% reduction of NO-stimulated GC activity (DEA-NO, 100 µM) in renal homogenates with the residual NO-GC activity reflecting the content of the NO-GC2 isoform (0.5 ± 0.1 nmol/mg min versus 2.3 ± 0.3 nmol cGMP/mg min in WT; Figure 1A). The results indicate that NO-GC1 amounts to 80% and the NO-GC2 to 20% of the total NO-GC content in kidney. Measurements of cGMP hydrolysis revealed that PDE activity (1µM cGMP) is unaltered in NO-GC1-deficient kidneys (KO 527 ± 47 versus WT 517 ± 87 pmol GMP/mg min). 

Analysis of the protein content in Western blot revealed that the loss of the α_1_ subunit in kidney was accompanied by a decrease of the β_1_ subunit content (60 ± 16%; Figure 1B). The observed reduction of the β_1_ subunit confirms the NO-GC1 (α_1_β_1_ heterodimer) as the major NO-GC isoform in kidney. In addition, similar amounts of the α_2_ subunit were determined in WT and NO-GC1 KO kidneys excluding upregulation of the NO-GC2 isoform (α_2_β_1_ heterodimer).

Compared to NO-GC activity, the cGMP content in renal cortical slices of NO-GC1 KO mice was reduced by only 45% compared to WT under untreated conditions (KO 0.27 ± 0.04 pmol cGMP/mg protein, versus WT 0.5 ± 0.07 pmol cGMP/mg protein; Figure 1C). In WT slices, the inhibitor of NO-stimulated GC activity, ODQ (20 µM, 15 min), reduced the cGMP content by 56% indicating continuous eNOS activation in renal slices under basal i.e. untreated conditions. In NO-GC1 KO slices, ODQ treatment did not lower the cGMP content showing that in the absence of NO-GC1, cGMP formation in response to basically produced NO is too low to yield measurable cGMP increases.

To determine the capacity for renal cGMP formation, we incubated the renal cortical slices with exogenous NO (DEA-NO 100 µM, 3 min) or carbachol (100 µM, 3 min) which stimulates endogenous NO formation by eNOS. DEA-NO caused a 20-fold increase of the cGMP content in WT (10 ± 1.4 pmol cGMP/mg protein). The cGMP content in the NO-GC1 KO was also increased 20-fold by DEA-NO but was 50% lower than in WT (5 ± 0.7 pmol cGMP/mg protein). Carbachol caused a 7-fold and 2-fold cGMP increase in WT and NO-GC1 KO, respectively (WT 3.6 ± 0.4 versus KO 0.6 ± 0.2 pmol cGMP/mg protein). In sum, the decrease of the renal cGMP content was less than expected by the 80% reduction of the cGMP-forming enzyme.

### Renal vascular relaxation of NO-GC1 KO mice

Next, we wanted to know whether the reduced cGMP content in NO-GC1 KO mice affects renal hemodynamics. Therefore, renal vascular relaxation was measured in the isolated perfused kidneys. In NO-GC1 KO kidneys, endothelium-dependent relaxation (30 µM carbachol) was reduced by 36% compared to WT (Figure 1D). In accordance, maximal renal vascular relaxation of the NO-GC1 KO kidneys by GSNO (S-nitrosogluthathione) was reduced to 56% of WT and the concentration-response curve was shifted to the right yielding about 17-fold higher EC_50_ values (KO 10.16 ± 0.51 µM GSNO versus WT 0.60 ± 0.1 µM GSNO; Figure 1E). 

To determine the amount of NO formed in response to carbachol in the renal vasculature, the carbachol response (35% in NO-GC1 KO and 54% in WT) was plotted to the respective GSNO concentration-response curve and corresponded to 7 µM GSNO in NO-GC1 KO kidneys and 0.6 µM GSNO in WT kidneys (see Figure 1E). These data show that relaxation in the NO-GC1 KO requires 10 times more NO than in WT kidneys and indicate that carbachol elicits a higher NO production in NO-GC1 KO kidneys than in WT to achieve the observed relaxation. 

To support the finding of an enhanced renal NO production in response to carbachol in NO-GC1 KO mice, expression of endothelial NO synthase (eNOS) was quantified by Western blot analysis. As shown in Figure 1F, expression of eNOS was 1.3-fold increased in NO-GC1 KO kidneys substantiating the notion of enhanced NO production in the NO-GC1 KO kidneys. To quantify NO production in the NO-GC1 KO kidneys more directly, NOS activity in renal homogenates was assessed in an enzymatic assay and monitored as NOS-dependent nitrosation of the NO-specific fluorescent probe DAF-FM diacetate [20]. NOS activity was found to be 4-fold higher in the NO-GC1 KO than in WT kidneys (KO 384 ± 116% versus WT 100 ± 20%; Figure S1). Specificity of the signal was controlled by adding the NOS inhibitor L-NAME. The data suggest that NO-GC1 deficiency in the renal vasculature is partially compensated by increased NO generation.

### Comparable blood pressures increase in NO-GC1 KO and WT mice in the 2K1C model

To study the NO/cGMP signaling under pathophysiological conditions, we challenged the NO-GC1 KO mice by the 2K1C operation which provokes renovascular hypertension by activation of the renin-angiotensin system. 

Four weeks after clipping the left renal artery (2K1C operation) in NO-GC1 KO and WT mice, the clipped kidneys exhibited a similar degree of atrophy, while right kidneys of both groups showed comparable compensatory hypertrophy (Table 1). The similar changes in renal weights observed in both groups indicated that the 2K1C-induced ischemic stress was comparable. Systolic blood pressures (SBP) measured by the tail-cuff method were elevated in 2K1C-operated NO-GC1 KO and WT mice (see Table 1). Interestingly, despite the absence of NO-GC1, blood pressures increases did not differ between KO and WT mice indicating that reduced cGMP levels did not aggravate hypertension in this model. 

**Table 1:** Effects of 2K1C operation on WT and NO-GC1 KO mice.

		**WT mice**			**NO-GC1 KO mice**	
		Sham	2K1C		Sham	2K1C
SBP (mmHg)		116 ± 2	128 ± 2^*^		116 ± 1	128 ± 2^*^
Kidney left (mg/g BW)		7.73 ± 0.29	4.03 ± 0.66^*^		7.50 ± 0.37	3.37 ± 0.59^*^
Kidney right (mg/g BW)		7.66 ± 0.33	8.54 ± 0.38		8.00 ± 0.41	8.81 ± 0.41

Data are means ± SEM (Blood pressure was measured in 10 WT and 11 KO mice; kidney weights were averaged over 18 WT and 11 KO mice per treated group). Body weight and heart weight were not affected by the 2K1C operation (data not shown). BW indicates body weight; * P<0.001 2K1C- vs. sham-operated mice of both groups

### The 2K1C operation reduces renal vascular relaxation in WT but not in NO-GC1 KO mice

**Figure 2:**
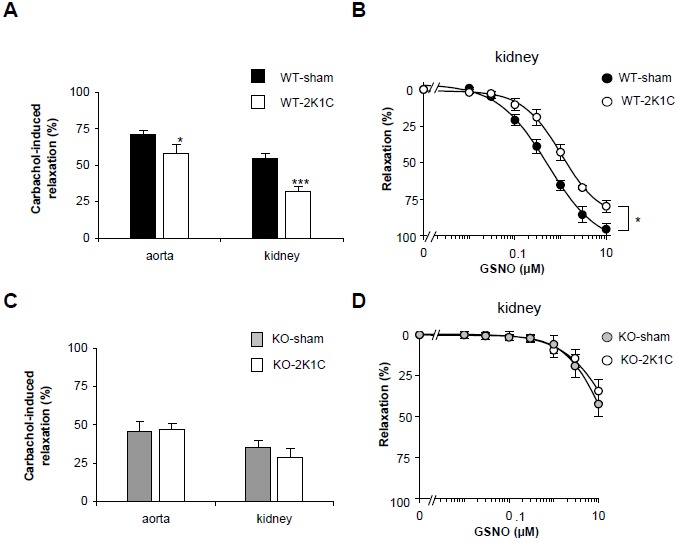
2K1C operation did not reduce vascular relaxation in NO-GC1 KOs but in WT mice. **A**, endothelium-dependent relaxation induced by carbachol (30 µM) in NA-contracted aortic rings and isolated perfused kidneys of sham- and 2K1C-operated WT mice (36 aortic rings of n=9 mice per group; kidneys of n=21 and 8 mice, respectively). * P< 0.05 versus aorta of WT-sham, paired 2-tailed Student's t test; ** P<0.001 versus kidney of WT-sham, unpaired 2-tailed Student's t test. **B**, concentration-response curves for GSNO-induced relaxation determined in NA-contracted isolated perfused kidneys of sham- and 2K1C-operated WT mice in the presence of L-NAME (n=5 and 7 mice, respectively). P=0.037, ANOVA for repeated measurements. **C**, endothelium-dependent relaxation induced by carbachol (30 µM) in NA-contracted aortic rings and isolated perfused kidneys of sham- and 2K1C-operated NO-GC1 KO mice (28 aortic rings of n=7 mice per group; kidneys n=12 and 7 mice, respectively). **D**, concentration-response curves for GSNO-induced relaxation determined in NA-contracted isolated perfused kidneys of sham- and 2K1C-operated NO-GC1 KO mice in the presence of L-NAME (n=8 and 6 mice, respectively). Experiments were performed using the non-clipped kidney of 2K1C mice and the respective kidney of sham mice.

The 2K1C operation has been published to reduce endothelium-dependent relaxation of aortic rings [13–16]. In accordance to these studies, we observed a reduction in endothelium-dependent relaxation of aortic rings in 2K1C-operated WT mice (14% of sham; Figure 2A). This reduction was found to be even more pronounced in the vasculature of the 2K1C WT kidneys (41% of sham; see Figure 2A). In order to differentiate between endothelium- and smooth muscle-dependent relaxations, we also used the NO donor GSNO. Compared to sham-operated WT mice, GSNO shifted the concentration-response curve to higher EC_50_ values in the kidneys of the 2K1C WT (EC_50_ 2K1C 1 ± 0.3 µM versus sham 0.5 ± 0.07 µM; Figure 2B) demonstrating a reduced NO sensitivity rather than a reduced NO generation in kidneys of 2K1C WT mice. 

In the NO-GC1 KO mice, the 2K1C operation did neither affect relaxation of aortic rings nor did it alter relaxation of isolated perfused kidneys (Figure 2C, D). NO-GC1 KOs have lower cGMP formation which found to be partially compensated by higher NO levels than in WT. Thus, the lack of difference between sham and 2K1C NO-GC1 KO further supports the notion that the reduced vascular relaxation in 2K1C WT mice depends on cGMP levels and not on the amount of NO. 

### The 2K1C operation does neither alter cGMP synthesis nor cGMP sensitivity

**Figure 3:**
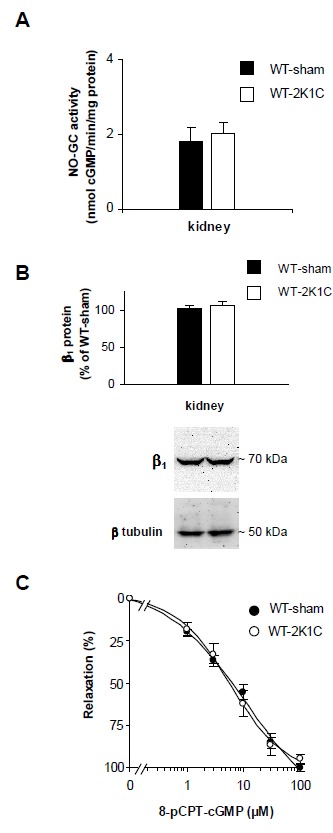
2K1C operation does not alter cGMP synthesis or cGMP sensitivity in WT kidneys. **A**, NO-stimulated cGMP-forming activity (DEA-NO 100 µM) determined in kidney homogenates of sham- and 2K1C-operated WT mice (n=6 mice per group). **B**, quantification of the β_1_ subunit content with respect to the subunit amount in sham-operated WT mice using subunit specific antibodies (n=12 mice per group). **C**, concentration-response curves for 8-pCPT-cGMP of NA-contracted isolated perfused kidneys of sham- and 2K1C-operated WT mice in the presence of L-NAME (n=13 and 7 mice, respectively). Experiments were performed using the non-clipped kidney of 2K1C mice and the respective kidney of sham mice.

To assess whether impaired cGMP synthesis accounts for the impaired vasodilator response to GSNO in 2K1C-operated WT mice, NO-stimulated cGMP-forming activities were determined in kidney homogenates. As shown in Figure 3A, NO-stimulated cGMP-forming activities did not differ between 2K1C-operated and control WT mice (2K1C, 2 ± 0.3 versus sham, 1.8 ± 0.3 nmol/mg min). In addition western blot quantification of the β_1_ subunit which is present equally in the NO-GC1 (α_1_β_1_) and NO-GC2 (α_2_β_1_) heterodimer did not reveal any differences between both groups (Figure 3B). Thus, we conclude that the 2K1C operation did not alter the expression of NO-GCs. Next, sensitivity towards cGMP was studied with the direct PKGI activator, 8-(p-chlorophenylthio)-cGMP (8-pCPT-cGMP). Renal relaxation in 2K1C-operated WT mice induced by the membrane-permeable cGMP analogue did not differ from that in control kidneys demonstrating that sensitivity towards cGMP was unchanged (Figure 3C).

### The 2K1C-induced reduction of vascular NO sensitivity is restored by PDE5 inhibition

**Figure 4:**
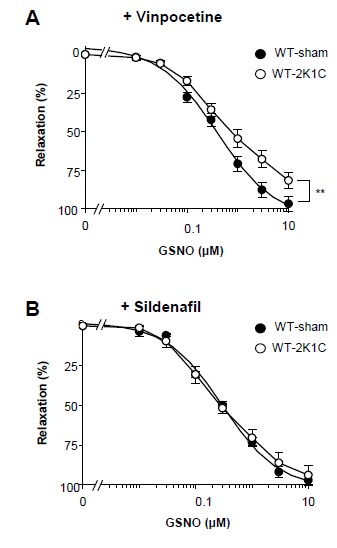
PDE5 inhibitor, sildenafil, restores reduced NO sensitivity of renal vessels from operated WT. Concentration-response curves for GSNO-induced relaxation determined in NA-contracted isolated perfused kidneys of sham- and 2K1C-operated WT mice in the presence of (**A**) the PDE1 inhibitor vinpocetine (10 µM; n=6 and 5 mice, respectively) or (**B**) the PDE5 inhibitor sildenafil (300 nM; n=5 mice per group). **P< 0.01, ANOVA for repeated measurements. Experiments were performed using the non-clipped kidney of 2K1C mice and the respective kidney of sham mice.

Subsequently, we asked whether enhanced cGMP degradation accounts for the reduction of NO sensitivity in 2K1C WT mice and used vinpocetine (10 µM) and sildenafil (0.3 µM) to inhibit PDE1 and PDE5, the main cGMP-hydrolyzing enzymes in smooth muscle. The PDE inhibitors were administered in concentrations which alone did not affect vascular reactivity in WT. Under PDE1 inhibiting conditions, the NO response in the non-clipped kidneys of 2K1C WT mice remained significantly reduced (Figure 4A). In contrast, inhibition of PDE5 restored the reduced NO sensitivity in 2K1C WT kidneys as sildenafil shifted the GSNO concentration-response curve to that determined in control kidneys in the presence of sildenafil (Figure 4B). These findings indicate a role of PDE5 in the reduction of vascular relaxation caused by the 2K1C operation.

### PDE5 is not up-regulated by the 2K1C operation

**Figure 5:**
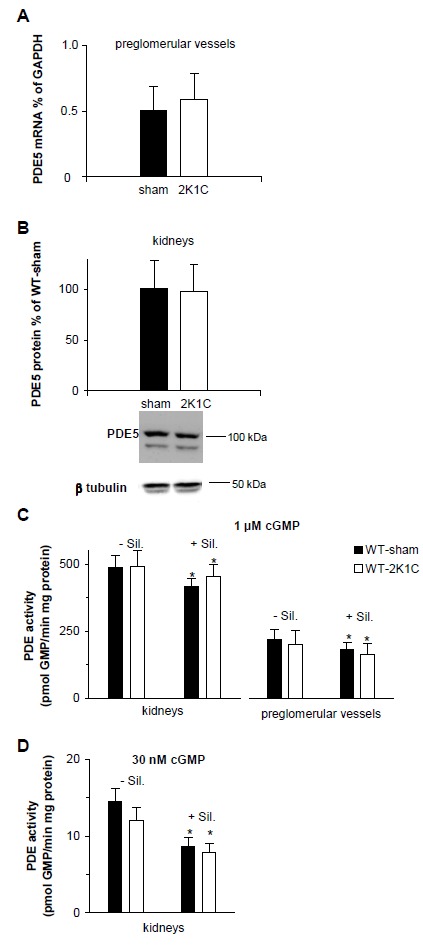
2K1C operation did not up-regulate PDE5. **A**, PDE5 mRNA in preglomerular vessels of sham- and 2K1C-operated WT mice detected by quantitative real-time PCR and quantified relative to GAPDH with the ∆C_T_ method (n=8 and 9 mice, respectively). **B**, PDE5 protein detected by Western blot in 50 µg kidney homogenates of sham- and 2K1C-operated WT mice and expressed as % of PDE5 content in kidneys of sham-WT mice (n=7 mice per group). A representative strip with PDE5 and the respective β tubulin bands of the same lanes is shown below. PDE activities in homogenates of kidneys and preglomerular vessels of sham- and 2K1C-operated WT mice in the absence and presence of sildenafil (100 nM) measured by 1 µM (**C**) or 30 nM cGMP (**D**). PDE activities were obtained of 3 mice per group in kidneys by 1 µM cGMP; n=13 and 10 mice per group, preglomerular vessels; n=6 per group kidneys by 30 nM. * P< 0.001 versus corresponding PDE activity without sildenafil, paired 2-tailed Student's t test. Experiments were performed using the non-clipped kidney of 2K1C mice and the respective kidney of sham mice.

To detect a possible PDE5 up-regulation induced by the 2K1C operation, we examined PDE5 mRNA and protein expression by quantitative real-time PCR and Western blot analysis in renal tissues. As shown in Figure 5A, levels of PDE5 mRNA were comparable in isolated preglomerular arterioles of 2K1C- compared to sham-operated mice. In accordance, PDE5 protein levels determined in kidney homogenates did not show any difference (Figure 5B). Together these results argue against an up-regulation of PDE5 as the reason for the reduced vascular relaxation in the 2K1C model.

### Greater effect of sildenafil in 2K1C WT mice points to an activated PDE5 state

PDE5 is known to exist in two different states, a non-activated and an activated state with an increased catalytic rate due to allosteric binding of cGMP to PDE5's GAF domains. To directly address activated PDE5 in 2K1C WT mice, we determined PDE activity which should be enhanced in the case of PDE5 activation. PDE activity was measured as cGMP hydrolysis in the presence and absence of sildenafil (100 nM) in kidneys and preglomerular arterioles of sham- and 2K1C-operated WT mice. Hence, we did not detect enhanced PDE5 or total PDE activity in kidneys or preglomerular arterioles of 2K1C-operated WT mice (Figure 5C and 5D). However, measurements revealed that PDE5 represents only a fractional amount of total PDEs (20% using 1 µM cGMP or 40% with 30 nM cGMP as substrate) and that PDE activity varies ~30% among mice. Thus, PDE5 activation may not be detectable in tissues with various PDE isoforms like the kidney. 

**Figure 6:**
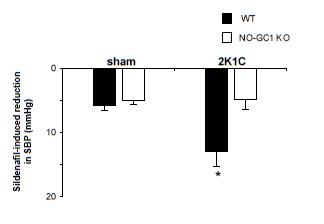
Sildenafil effects on blood pressures. Sildenafil (100 mg/kg d) effects on systolic blood pressures measured in conscious sham- and 2K1C-operated WT (WT-sham 118 ± 1 mmHg without, and 112 ± 1 mmHg with sildenafil; WT-2K1C 130 ± 4 mmHg without, and 118 ± 2 mmHg with sildenafil) and NO-GC1 KO mice (KO-sham 118 ± 1 mmHg without, and 113 ± 2 mmHg with sildenafil; KO-2K1C 125 ± 2 mmHg without, and 121 ± 2 mmHg with sildenafil) using tail-cuff manometer (n=4 WT-sham, 5 WT-2K1C, 6 KO-sham, and 10 KO-2K1C mice). Sildenafil administration and SBP measurements were performed during the 4^th^ week after operation. *P< 0.05 2K1C WT compared to sham WT, unpaired Student's t test. SBP indicates systolic blood pressure.

In an attempt to demonstrate the impact of putative higher PDE5 activity in the 2K1C WT mice *in vivo*, we treated 2K1C- and sham-operated WT and KO mice with sildenafil (100 mg/kg d for 1 week, three weeks after the 2K1C operation) and subsequently, monitored blood pressures in conscious mice by tail-cuff. As shown in Figure 6, sildenafil reduced blood pressures more efficiently in 2K1C-operated WT compared to sham WT mice (2K1C WT 13 ± 2 mmHg versus sham WT 6 ± 1 mmHg; p= 0.026) indicating a higher impact for sildenafil inhibition in the 2K1C model. In the NO-GC1 KOs, the sildenafil effect on blood pressure was comparable in operated and non-operated mice (2K1C KO 5 ± 2 mmHg versus sham KO 5 ± 1 mmHg) indicating that the cGMP-dependent activation of PDE5 does not occur in the NO-GC1 KO. 

### Enhanced eNOS-catalyzed NO formation induced by the 2K1C operation

**Figure 7:**
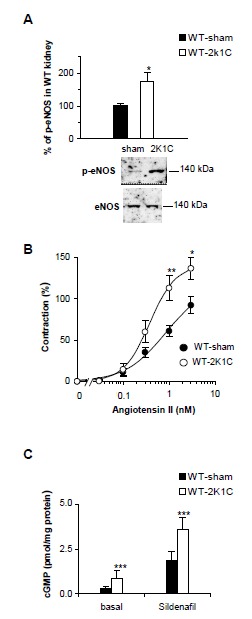
Evidence of enhanced eNOS-catalyzed NO formation induced by the 2K1C operation. **A**, Western blot detection of p-eNOS (serine 1177) and eNOS in 50 µg kidney homogenates of sham- and 2K1C-operated WT mice and quantification with respect to the ratio of p-eNOS to eNOS in kidneys of sham-operated WT (n=8 and 7 mice, respectively). * P< 0.05 versus WT-sham, unpaired Student's t test. **B**, concentration-response curves for angiotensin II-induced vasoconstriction of isolated perfused kidneys of sham- and 2K1C-operated WT mice in the presence of L-NAME (n=9 and 6 mice, respectively). * P< 0.05, and ** P< 0.01 versus WT-sham, unpaired Student's t test. **C**, cyclic GMP content determined in renal cortical slices of sham- and 2K1C-operated WT mice without any addition (15 slices of 5 mice per group) or in the presence of sildenafil (100 µM, 10 min; 15 slices of 5 mice per group). ***P< 0.001 versus WT-sham, unpaired Student's t test. Experiments were performed using the non-clipped kidney of 2K1C mice and the respective kidney of sham mice.

An activated state of PDE5 implies enhanced NO/cGMP formation in the 2K1C model. Thus, we used Western-blot analysis to determine the eNOS content in the 2K1C WT kidneys. While eNOS expression was unaltered, increased phosphorylation of eNOS on Ser-1177, a marker for increased eNOS activity was detected (Figure 7A). Phosphorylation of eNOS depends on shear stress which is provoked by vasoconstriction. In accordance, we found an increased contractile response towards Angiotensin II in 2K1C WT mice explaining the enhanced eNOS activation (Figure 7B). 

To confirm our assumption of an increased biologically active NO in 2K1C WT mice, we measured cGMP levels in renal cortical slices of sham- and 2K1C-operated WT mice (Figure 7C). Indeed, basal levels of cGMP were found to be 2.7-fold higher in kidneys of 2K1C WT mice than in the control group (2K1C, 0.84 ± 0.08 versus sham, 0.31 ± 0.06 pmol cGMP/mg protein; p< 0.001). Furthermore, cGMP accumulation in the presence of sildenafil (100 µM, 10 min) was higher in kidneys of 2K1C WT mice. In sum, our results indicate enhanced eNOS-catalyzed NO formation in 2K1C WT mice and support our hypothesis that enhanced NO/cGMP causing PDE5 activation finally results in a decrease of NO sensitivity. 

## Discussion

The NO/cGMP signaling has an established role in vascular relaxation and regulation of blood pressure. Genetic or pharmacological inhibition of the cascade decreases relaxation and increases blood pressure [23,24]. In the vasculature, two different forms of the cGMP forming NO-GCs, NO-GC1 and NO-GC2, exist which correspond to the earlier described α_1_β_1_ and α_2_β_1_ heterodimer, respectively. We previously reported that deletion of the NO-GC1 resulted in 90% reduction of the total NO-GC content in aortic tissues, and an attenuated endothelium- und smooth muscle-dependent relaxation. 

Here we describe that in kidneys of NO-GC1 KO mice, NO-stimulated cGMP formation is also markedly reduced (80% of WT) with the amount of the remaining NO-GC2 being higher in kidney (0.5 ± 0.1 nmol cGMP/mg min) than in aorta (0.2 ± 0.1 nmol cGMP/mg min). A particular role for NO-GC2 in the renal vasculature is supported by the ability of NO to increase cGMP levels in the NO-GC1 KO renal cortical slices while in aorta NO stimulation did not yield measurable cGMP increases. On the other hand it should be kept in mind that the PDE isoforms expressed in a certain tissue have a great impact on the cGMP response. 

Relaxation of renal vasculature was determined in isolated perfused kidneys, a model for examining the function of resistance arteries. In accordance with the reduced cGMP formation, relaxation was attenuated in NO-GC1 KO mice. The residual NO/cGMP effects are mediated by NO-GC2 demonstrating that cGMP produced by this isoform regulates physiological processes. Hence, analysis of the carbachol- and GSNO-induced relaxation revealed an increase in NO production in responses to carbachol in the NO-GC1 KO. In line with this observation, up-regulation of eNOS expression was found in Western blot analysis. Thus; we conclude that enhanced NO formation supports NO-GC2-catalyzed cGMP formation in the NO-GC1 KO mice to ensure an adequate renal perfusion. 

Despite the reduction of the cGMP-forming enzyme, the NO-GC1 KO mice are not hypertensive. To provoke hypertension, we challenged NO-GC1 KO mice with the 2K1C operation, which activates the renin-angiotensin-aldosterone system (RAAS). The 2K1C operation increased blood pressures to a similar extend in NO-GC1 KO and WT mice showing that the degree of hypertension in the 2K1C model is not influenced by the level of NO/cGMP. This is surprising at first, but in line with the finding that the 80-90% reduction of the cGMP forming enzyme in the NO-GC1 KO does not affects blood pressure (on the C57Bl/6 background) whereas additional deletion of the remaining enzyme (NO-GC2) results in pronounced hypertension [24]. The results indicate that only minor amounts of cGMP (as supplied by the NO-GC2) are sufficient to prevent blood pressure increases. Hypertension of the NO-GC1 KO mice described by Buys and colleagues was found to be strain-specific and dependent on higher activity of the RAAS in the 129S6 background [7]. 

The 2K1C operation has been reported to reduce endothelium-dependent relaxation of aortic rings [13–16]. Accordingly, 2K1C-operated WT mice showed a reduction of carbachol-induced relaxation which was more pronounced in kidneys than in aortas. In contrast, carbachol-induced relaxation was not reduced in the 2K1C-operated NO-GC1 KO mice. Kidneys of operated WT mice also exhibited a reduced sensitivity to exogenous NO, thus, the attenuated responsiveness towards NO most likely accounts for the reduction of endothelium-dependent relaxation. This result is in conflict with reports by others who propose endothelial dysfunction i.e. decreased NO bioavailability as the major reason for the reduction of endothelium-dependent relaxation in renovascular hypertension [13–16]. On the other hand, the discrepancy may be explained by different vessels used: whereas in our study renal resistance arteries responsible for blood pressure regulation were analyzed, large conductance vessels were studied by the colleagues. 

In search for the reason of the reduced NO sensitivity in 2K1C-operated WT mice NO-stimulated GC activity was measured in kidney homogenates. Similar rates of cGMP synthesis in control and 2K1C-operated mice ruled out differences in NO-GC expression. Also relaxation induced by the cGMP analogue 8-pCPT-cGMP was unaltered in the 2K1C-operated WT mice and we conclude that sensitivity or activity of the cGMP-dependent protein kinases is unchanged. 

Next, we addressed a possible increase of cGMP degradation in vascular smooth muscle by analyzing NO-induced relaxation in the presence of PDE1 or PDE5 inhibitors. As PDE1 is stimulated by Ca^2+^, the enzyme was a tempting candidate to mediate an AngII-induced decrease of relaxation [25,26]. However, inhibition of PDE1 did not improve sensitivity to exogenous NO. Unexpectedly, PDE5 inhibition restored the reduced NO sensitivity observed in 2K1C WT pointing to PDE5 as the responsible component for the reduction of vascular relaxation in renovascular hypertension. The mechanism through which PDE5 is affected in renovascular hypertension is not clear. Although, it has been reported that Ang II upregulates PDE5 in VSMCs [27], we did not detect any changes in PDE5 protein or mRNA levels in kidneys from 2K1C WT mice. 

Within NO/cGMP signaling, a negative feedback regulation depending on PDE5 activation has been reported [10,12]. In this context, cGMP does not only bind to the catalytic center but also binds to PDE5's regulatory GAF-A domains thereby enhancing catalytic rate [10,11]. Thus, increased NO/cGMP signaling finally leads to its desensitization. However, the detection of an activated cGMP-bound PDE5 state ex vivo is difficult as the cGMP used as substrate in PDE activity measurements will also shift non-activated PDE5 to the activated state. The fact, that the 2K1C-operation did not reduce vascular relaxation in the NO-GC1 KO mice, indicates that here NO/cGMP signaling is too low to initiate internal feedback regulation.

In order to confirm the impact of activated PDE5 in renovascular hypertension we used an in vivo approach, where we treated mice with sildenafil. As expected for an activated PDE5, sildenafil reduced blood pressure more efficiently in 2K1C- than in sham-operated WT mice. In line, the sildenafil effect on blood pressure was not increased by the operation in the NO-GC1 KOs. PDE5 activation requires enhanced NO/cGMP signaling. In support of this notion, increased renal contractility, eNOS phosphorylation at serine1177 and cGMP levels were found in the operated WT mice. Increased NO production in the 2K1C model has also been postulated by others [28–30].

Our results support the following scenario: renovascular hypertension induced by the 2K1C operation enhances NO/cGMP signaling in the kidney finally resulting in long-lasting PDE5 activation which in turn is responsible for the reduced NO/cGMP-mediated relaxation. In line, reduction in vascular relaxation does not occur in NO-GC1-deficient mice as cGMP forming activity is lower and long-lasting PDE5 activation does not happen.

According to our concept, low doses of PDE5 inhibitors reported to preferentially inhibit the cGMP-bound, activated state of the enzyme, appear to be indicated as a possible rational pharmacological intervention in the treatment of renovascular hypertension.

## Supporting Information

Figure S1 **Enhanced NOS activity in kidneys of NO-GC1 KO mice.** NOS activity in kidney homogenates of WT and NO-GC1 KO mice determined as NOS-dependent nitrosation of the NO-specific fluorescent probe DAF-FM diacetate (n=5 and 4 mice, respectively). * P< 0.05 versus WT, unpaired Student's t test.
(TIF)
