# Supplementary figures and images for: Phosphodiesterase 5 Attenuates the Vasodilatory Response in Renovascular Hypertension
Source: PLoS One. 2013 Nov 15;8(11):e80674. doi: 10.1371/journal.pone.0080674 (PMC3829872; doi:10.1371/journal.pone.0080674)

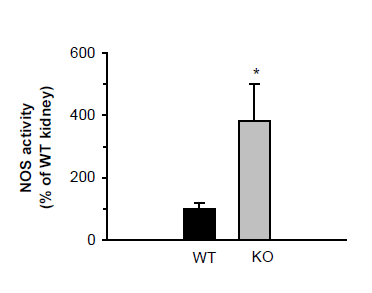

Supplement: Figure S1 — Enhanced NOS activity in kidneys of NO-GC1 KO mice. NOS activity in kidney homogenates of WT and NO-GC1 KO mice determined as NOS-dependent nitrosation of the NO-specific fluorescent probe DAF-FM diacetate (n=5 and 4 mice, respectively). * P< 0.05 versus WT, unpaired Student's t test. (TIF) [file pone.0080674.s001.tif]
